# Standardized butanol fraction of WIN-34B suppresses cartilage destruction via inhibited production of matrix metalloproteinase and inflammatory mediator in osteoarthritis human cartilage explants culture and chondrocytes

**DOI:** 10.1186/1472-6882-12-256

**Published:** 2012-12-15

**Authors:** Jeong-Eun Huh, Byung-Kwan Seo, Yong-Hyeon Baek, Sanghoon Lee, Jae-Dong Lee, Do-Young Choi, Dong-Suk Park

**Affiliations:** 1 Oriental Medicine Research Center for Bone & Joint Disease, East–west Bone & Joint Research Institute, Kyung Hee University, 149, Sangil-dong, Gangdong-gu, Seoul 134-727, Republic of Korea; 2 Department of Acupuncture & Moxibustion, Kyung Hee University Hospital at Gangdong, 149, Sangil-dong, Gangdong-gu, Seoul, 134-727, Republic of Korea; 3 Department of Acupuncture & Moxibustion, College of Oriental Medicine, Kyung Hee University, 1 Hoegi-dong, Dongdaemun-gu, Seoul, 130-702, Republic of Korea

**Keywords:** WIN-34B, Standard compounds, Cartilage protection, Matrix proteinases, Inflammatory mediators

## Abstract

**Background:**

WIN-34B is a novel Oriental medicine, which represents the *n*-butanol fraction prepared from dried flowers of *Lonicera japonica* Thunb and dried roots of *Anemarrhena asphodeloides* BUNGE. The component herb of WIN-34B is used for arthritis treatment in East Asian countries. The aim of this study was to determine the cartilage-protective effects and mechanisms of WIN-34B and its major phenolic compounds, chlorogenic acid and mangiferin, in osteoarthritis (OA) human cartilage explants culture and chondrocytes.

**Methods:**

The investigation focused on whether WIN-34B and its standard compounds protected cartilage in interleukin (IL)-1β-stimulated cartilage explants culture and chondrocytes derived from OA patients. Also, the mechanisms of WIN-34B on matrix metalloproteinases (MMPs), tissue inhibitor of matrix metalloproteinases (TIMPs), inflammatory mediators, and mitogen-activated protein kinases (MAPKs) pathways were assessed.

**Results:**

WIN-34B was not cytotoxic to cultured cartilage explants or chondrocytes. WIN-34B dose-dependently inhibited the release of glycosaminoglycan and type II collagen, increased the mRNA expression of aggrecan and type II collagen, and recovered the intensity of proteoglycan and collagen by histological analysis in IL-1β-stimulated human cartilage explants culture. The cartilage protective effect of WIN-34B was similar to or better than that of chlorogenic acid and mangiferin. Compared to chlorogenic acid and mangiferin, WIN-34B displayed equal or greater decreases in the levels of MMP-1, MMP-3, MMP-13, ADAMTS-4, and ADAMTS-5, and markedly up-regulated TIMP-1 and TIMP-3. WIN-34B inhibited inflammatory mediators involved in cartilage destruction, such as prostaglandin E2, nitric oxide, tumor necrosis factor-alpha, and IL-1β. The phosphorylation of extracellular signal-regulated kinase, c-Jun N-terminal kinase (JNK), and p38 was significantly reduced by WIN-34B treatment, while phosphorylation of JNK was only inhibited by chlorogenic acid or mangiferin in IL-1β-stimulated chondrocytes.

**Conclusions:**

WIN-34B is potentially valuable as a treatment for OA by virtue of its suppression of MMPs, ADAMTSs, and inflammatory mediators, and it’s up-regulation of TIMP-1 and TIMP-3 involved in the MAPK pathway.

## Background

Osteoarthritis (OA) is a multifactorial degenerative joint disease in which the cartilaginous matrix of the articular joint is destroyed. The anabolic and catabolic imbalance in articular cartilage plays a crucial role in OA pathogenesis. As a result, enhanced degradation occurs in the macromolecular components including aggrecan and collagen [1]. The superficial zone of OA cartilage, which is characterized by degenerative changes (fibrillations, chondrocyte clusters, and degenerative matrix changes) contains interleukin (IL)-1β, tumor necrosis factor-alpha (TNF-α), and matrix metalloproteinases (MMPs) including MMP-1, 2, 3, 8, 9, and 13 [2]. IL-1β and TNF-α can induce chondrocytes to produce other cytokines as well as stimulate catabolic proteinases (MMPs or aggrecanases) and proinflammatory mediators such as nitric oxide (NO) and prostaglandin E_2_ (PGE_2_) [3]. In this way, they can alter compensatory biosynthetic homeostasis and, in turn, break down the integrity of the extracellular matrix (ECM) [4-6].

The disease progression and structural changes show that the release of sulfated glycosaminoglycan (GAG), the degradation of type II collagen, and the over-production of cytokines are central pathophysiological events in OA. The course of the disease is related to a number of complex pathways and mechanisms, among which are the excessive production of proteolytic enzymes such as the aggrecanases and MMPs [4-6]. Aggrecan is degraded by both aggrecanases and MMPs, whereas type II collagen is degraded by MMPs [6]. With these protease activities in mind, it is logical to target these actions to stop the progression of cartilage degradation in OA.

IL-1β can induce chondrocytes to produce proinflammatory mediators such as PGE_2_ and NO, as well as stimulating catabolic proteinases (MMPs or aggrecanases) [1-3]. NO is a crucial mediator of the inflammatory response by virtue of its physiological effects and its ability to regulate the expression of inflammatory proteins [3]. In this way, they can alter compensatory biosynthetic homeostasis and break down the integrity of the ECM [4-7].

Recent studies have demonstrated that mitogen-activated protein kinases (MAPKs) play a key role in the cytokine regulation of MMP expression and consequent cartilage destruction [8]. In OA cartilage, the level of phosphorylated MAPKs, including extracellular signal-regulated kinase (ERK), c-Jun amino-terminal kinase (JNK), and p38 appears to be higher than that in normal cartilage [9]. MAPK pathways can be specifically activates downstream to over-production of MMP-1, -3, and −13, TNF-α, and NO [10-12]. However, the precise downstream mechanism is unknown, which limits effective therapeutic interventions in OA.

While various attempts have been made to develop disease-modifying OA drugs (DMOADs) that neutralize inflammatory cytokines and proteinases, enhance factors related to cartilage and bone homeostasis, and intervene in intracellular signaling pathways, the results to date have been unsatisfactory regarding the effects and safety of these drugs [13,14]. The combination of systemic and topical administration of non-steroidal anti-inflammatory drugs (NSAIDs), steroids, glucosamine, and opioids is generally prescribed for OA patients, but current treatment options for more effective and safer management of chronic OA are insufficient [15-17]. Along with the alleviation of clinical symptoms, more effective therapeutic strategies to prevent the progression of disease and aid in the recovery of tissue damage are needed [18]. As an alternative to the existing treatment, research into natural materials derived from herbs utilized for the safe cure for OA has been done.

To develop a novel OA treatment, we investigated the cartilage protection, analgesia, and anti-inflammation properties of 200 medicinal herbs used clinically for their anti-inflammatory and analgesic properties in traditional medicine. WIN-34B, a compound extracted from two herbs, the flowers of *Lonicera japonica* Thunb and roots of *Anemarrhena asphodeloides* BUNGE, was selected from the screen. WIN-34B demonstrated excellent anti-inflammatory, analgesic, and anti-osteoarthritic properties in the experimental animal models [19], and did not cause any acute or chronic toxicity or gastric mucosal damage in the animal models [20].

In this study, we investigated whether WIN-34B and its standard compounds have cartilage protective effects in IL-1β induced human cartilage explants culture. We assessed the viability of WIN-34B in the presence or absence of IL-1β-induced cartilage explants culture and chondrocytes, levels of GAG and type II collagen, histochemical features, levels of matrix proteinases [ADAMTSs, MMPs, and tissue inhibitors of matrix metalloproteinase (TIMPs)] and inflammatory mediators (PGE_2_, NO, IL-1β, and TNF-α) and the phosphorylation of MAPK signaling.

## Methods

### Preparation of WIN-34B

WIN-34B was prepared by extracting a mixture of two medicinal herbs (dried flowers of *Lonicera japonica* and dried roots of *Anemarrhena asphodeloides*) at a respective ratio of 2:1 (w/w) with 50% (v/v) ethanol for 4 h at 85°C. After the extracted solution was filtered and evaporated *in vacuo*, the resulting concentrate was dissolved in 225 ml of distilled water and partitioned with 195 ml of *n*-butanol. The *n*-butanol layer was evaporated *in vacuo* and lyophilized for complete removal of the residual solvent to give 11 g of brown powder, for a yield of 7%. WIN-34B was standardized for quality control according to a previous report [19].

### High performance liquid chromatography (HPLC) analysis of WIN-34B

**Figure 1:**
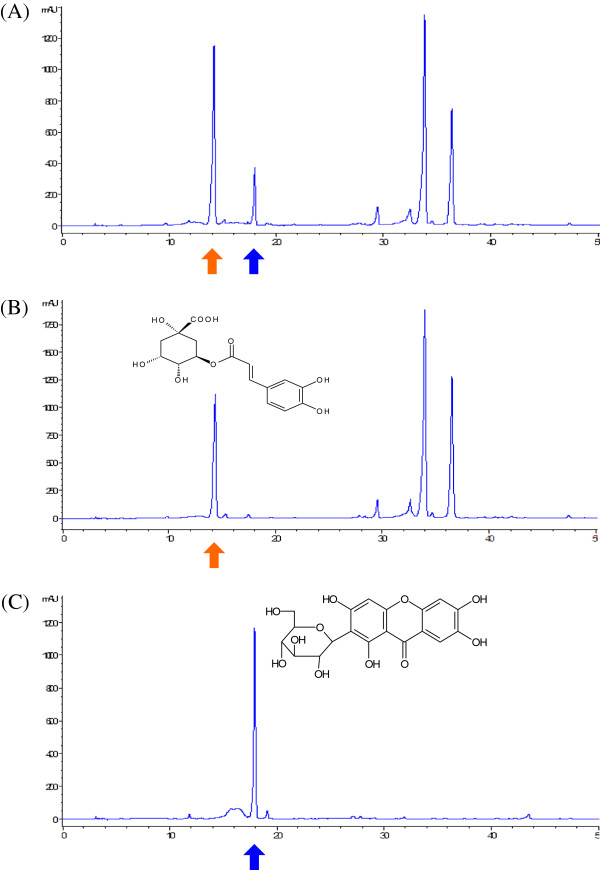
**Representative HPLC chromatogram of the extracts of WIN**-**34B and its standard compounds. **HPLC chromatogram of WIN-34B (**A**), major compound of chlorogenic acid (**B**) and mangiferin (**C**). Chlorogenic acid was detected at approximately 14 min and mangiferin was detected at approximately 18 min at 254 nm in this system.

WIN-34B was standardized for quality control according to a previous report [19], and used high pressure liquid chromatography (HPLC) analysis to identify the standard compounds, mangiferin and chlorogenic acid. Chromatographic analysis of WIN-34B was performed with a reverse-phase HPLC system (Waters, Milford, MA) equipped with the Waters Breeze System (2695 Separations Module, 996 Photodiode Array Detector, Empower 2 Software Build 2154). Separation was carried out using a YMC Hydrosphere C_18_ column (4.6 × 250 mm, particle diameter of 5 μm, YMC, Kyoto, Japan) at 30°C. The mobile phase consisted of 0.1% phosphoric acid solution in pump A (88.8%) and acetonitrile in pump B. Elution was undertaken using step gradients [B; 11.2-11.2% (0–16 min), 11.2-13.2% (16–17 min), 13.2-13.2% (17–28 min), and 13.2–100% (28–33 min)] at a flow rate of 1.0 ml/min. Detection of chlorogenic acid and mangiferin were performed at 327 nm and 254 nm, respectively (Figure 1).

### Cartilage explants culture

The collection of human OA cartilage was approved by medical ethical regulations of the Kyung Hee University Medical Center (KHMC-IACUC-2010011) and was obtained from the femoral chondyle and tibia plateau after nine patients undergoing total knee arthroplasty at the Kyung Hee University Medical Center provided consent. The average patient age was 62 years and patients included two males and seven females. NSAID medication was stopped 7 days before surgery and previous medication use was not expected to interfere with the studies. Two orthopedists read sites from all regions of the knee joint under a microscope. Only cartilage that appeared to be of full thickness with significant fibrillation was selected, so most joints appeared worse than the cartilage used here. Cartilage slices were aseptically cut as thick as possible from the articular bone surface, cut into square pieces, aseptically weighed (range 25 ± 0.1 mg), and cultured individually in 48-well plates with 400 μl of complete culture medium. The complete culture medium consisted of Dulbecco’s modified Eagle’s medium (DMEM) supplemented with 10 mM HEPES, penicillin (100 IU/ml), streptomycin (100 μg/ml), and 5% fetal bovine serum (FBS). After 24 h, the cartilage medium was changed to basal culture medium (DMEM supplemented with 10 mM HEPES, 100 IU/ml penicillin, 100 μg/ml streptomycin, and 2% FBS).

### WIN-34B treatment of human cartilage explants culture

Experimental groups consisted of IL-1β-unstimulated control group, IL-1β-treated group (10 ng/ml), IL-1β-treated group with WIN-34B (40, 100, 200 μg/ml), IL-1β-treated group with chlorogenic acid (CA; 40, 100, 200 μg/ml; Sigma-Aldrich, St. Louis, MO, USA), and IL-1β-treated group with mangiferin (MF; 40, 100, 200 μg/ml; Sigma-Aldrich). Cartilage pieces were placed in 48-well plates and treated with 10 ng/ml human recombinant IL-1β (R&D Systems, Minneapolis, MN, USA) in basal culture medium. After 1 h of pretreatment, WIN-34B, CA, or MF was added to the basal culture media and then cultures were incubated in a humidified 5% CO_2_ incubator at 37°C. Reagents were replaced every 3 days, and supernatants were harvested at 7, 14, and 21 days. Supernatants were stored at −20°C until assayed.

### Cytotoxicity assay

As an indicator of cytotoxicity, the cytoplasmic enzyme lactate dehydrogenase (LDH) was measured in the culture medium. An optimized LDH test (Promega, Madison, WI, USA) was used to quantify LDH activity in the medium of the cartilage explants culture.

### GAG degradation assay

The level of GAG in the cartilage explants culture medium at 7, 14, and 21 days was determined by measuring the amount of polyanionic material reacting with 1, 9-dimethylmethylene blue. Twenty microliter samples were mixed with 100 μl of DMB reagent (48 mg/ml DMB, 40 mM glycine, 40 mM NaCl, 10 mM HCl, pH 3.0) for 30 min at room temperature and quantified spectrophotometrically at 590 nm in a Spectramax apparatus (Molecular Devices, Sunnyvale, CA, USA). All measurements were performed in quadruplicate. Quantification was performed using a standard curve of chondroitin 6-sulfate from shark cartilage (Sigma-Aldrich) in the range of 0–35 μg/ml. Protein concentrations of the culture supernatants were also measured using the Bradford method (Pierce, Rockford, IL, USA) and then converted into μg/mg.

### Type II collagen degradation assay

Type II collagen levels in the medium of the cartilage explants culture at 7, 14, and 21 days was determined using the Sircol Type II Collagen Assay Kit (Biocolor, Carrickfergus, Northern Ireland). Samples (100 μl) were mixed with Sirius red dye containing sulfonic acid, which reacts specifically with the basic side chain groups of type II collagens, for 30 min at room temperature using a mechanical mixer. After centrifuging for 10 min at 12,000 rpm, the unbound dye was removed, and the dye bound to type II collagen was dissolved in 0.5 N NaOH. Absorbance was measured at 540 nm using a Spectramax ELISA reader (Molecular Devices). All measurements were performed in quadruplicate. Concentrations were calculated using a standard curve in the range of 0–200 μg/ml with standards provided by the manufacturer.

### Reverse transcriptase-polymerase chain reaction (RT-PCR)

Total RNA was extracted from OA cartilage explants by homogenizing with TRIzol reagent (Gibco Life Technologies, Grand Island, NY, USA) according to the manufacturer's instructions. Reverse transcription of the total RNA was carried out for 60 min at 42°C followed by 15 min at 72°C using an RT-PCR system (TaKaRa Bio, Seoul, Korea), which contained RT buffer, oligo (dT) 12-mer, 10 mM dNTP mix, 0.1 M dithiothreitol, reverse transcriptase, and RNase inhibitor. PCR using specific primers for each cDNA was carried out in a PCR reaction volume of 10 μl supplemented with 2.5 units of TaKaRa Taq™, 1.5 mM each dNTP, 1× PCR buffer, and 20 pmol of each primer (Table 1). After initial denaturation for 5 min at 95°C, 35 amplification cycles were performed for aggrecan, type II collagen, ADAMTS-4, ADAMTS-5, MMP-1, MMP-3, MMP-13, TIMP-1, and TIMP-3 (one cycle = 1 min at 95°C for denaturation, 1 min at 55°C for annealing, and 1.5 min at 72°C for extension), as well as for β-actin (one cycle = 1 min at 95°C for denaturation, 1 min at 58°C for annealing, and 1.5 min at 72°C for extension). After amplification, PCR products were separated by electrophoresis on 1.8% agarose gels and visualized using ethidium bromide staining and ultraviolet irradiation.

**Table 1:** **Primer Design for Quantitative RT**-**PCR Analysis**

**mRNA**	**Primers**^**a**^	**Annealing Tm **(**cycle**)
Aggrecan	Fw: 5^′^-TGAGGAGGGCTGGAACAAGTACC-3^′^	58°C (32)
	Rv: 5^′^-GGAGGTGGTAATTGCAGG GAACA-3^′^	
Col II	Fw: 5^′^-AACACTGCCAACGTCCAGAT-3’	58°C (32)
	Rv: 5^′^-CTGACGCACGGTATAGGTGA-3’	
ADAMTS-4	Fw: 5’-GTCTGTGTC CAGGGCCGATGC-3’	55°C (32)
	Rv: 5’-GCCGCCGAAGGATCTCCAGAA-3’	
ADAMTS-5	Fw: 5’-GCGGATGTGTGCAAGCTGACC-3’	55°C (32)
	Rv: 5’-AGTAGCC CATGCCATGCAGGA-3’	
MMP-1	Fw: 5’-TCAGTTCGTCCTCACTCCAG-3’	58°C (32)
	Rv: 5’-TTGGTCCACCTGTCATCTTC-3’	
MMP-3	Fw: 5’-ATGGACCTTCTTCAGCAA-3’	58°C (32)
	Rv: 5’-TCATTATGTCAGCCTCTC-3’	
MMP-13	Fw: 5’-AGGAGCATGGCGACTTCTAC-3’	58°C (32)
	Rv: 5’-TAA AAA CAG CTC CGC ATC AA-3’	
TIMP-1	Fw: 5’-GCA ACTCCGACCTTGTCATC-3’	58°C (32)
	Rv: 5’-AGCGTAGGTCTTGGTGAAGC-3’	
TIMP-3	Fw:5’-GGCGGCAGCAGCGGCAATGAC-3’	58°C (32)
	Rv:5’-TACCAGCTTCTTCCCCACCACCTT-3’	
β-actin	Fw: 5’-GCTCTCCAGAACATCACTCCTGCC-3’	58°C (27)
	Rv: 5’-CGTTGTCATACCAGGAAATGAGCTT-3’	

^a^Fw, forward; Rv, reverse; GAPDH, glyceraldehyde-3-phosphate dehydrogenase; Tm, temperature

### Histological analysis

Cartilage explants pieces were fixed in 10% neutral formalin, dehydrated with graded ethanol, embedded in paraffin, and sectioned into 4 μm-thick slices. Sectioned tissues were deparaffinized and stained with Safranin O and Masson's Trichrome to detect proteoglycan and collagen in the cartilage. The staining intensities of Safranin O and Masson's Trichrome were quantified by i-solution program (IMT i-solution, Seoul Korea) after capture using an Axiocam MRc5 CCD camera (Carl Zeiss, Jena, Germany) at x40 magnification on histologic sections. A pathologist with no prior knowledge of the test reagents examined the stained slides.

### Enzyme-linked immunosorbant assay (ELISA)

The levels of MMP-1, MMP-3, MMP-13, TIMP-1, TIMP-3, IL-1β, and TNF-α in conditioned media from OA cartilage explants at 7 days were measured using human ELISA kits (R&D Systems), according to the manufacturer's instructions.

### Aggrecanase activity assay

Conditioned medium in cartilage explants at 7 days from the onset of culture was incubated in the presence of 1% w/v bovine serum albumin (BSA) in phosphate buffered saline (PBS)/Tween 20 (0.05% v/v) for 2 h at 25°C on a 96-well plate (Biosource) containing a monoclonal antibody that recognizes KS chains and, according to the manufacturer, is not impacted by other non-KS glycosaminoglycans, including hyaluronic acid, chondroitin sulfate, and heparin sulfate. Fragments containing ARGSVIL neoepitope were detected using biotinylated monoclonal antibody (mAb) OA-1 (PIERCE, Rockford, IL, USA). Levels of bound biotinylated mAb OA-1 were detected using 1 μg/ml streptavidin-horseradish peroxidase (HRP) and TMB as a substrate. Absorbance was determined following acidification using a microplate reader at a wavelength of 450 nm. Calibration curves for standard ARGSVIL peptide were run in parallel, and the amounts of ARGSVIL peptide produced in hydrolytic reactions were calculated from the calibration curves.

### Measurement of PGE_2_

PGE_2_ production was determined from the supernatant of cultured OA cartilage explants at 7 days using assay kits (R&D Systems) performed per the manufacturer’s instructions.

### Measurement of NO

NO synthesis was determined from the supernatant of cultured OA cartilage explants at 7 days by colorimetric assay as an indicator of NO production. Briefly, a 100 μl aliquot of medium was mixed with 100 μl of Greiss reagent (1% sulfanilamide in 5% phosphoric acid and 0.1% naphthylethylenediamine dihydrochloride in water) (Sigma-Aldrich) in flat-bottom, 96-well immunoassay plates (SPL Life Sciences, Seoul, Korea). After incubating for 10 min at room temperature, absorption was measured at 550 nm with a Spectra Max 340 multichannel spectrophotometer (Molecular Devices). The nitrite concentration was determined from a standard curve generated using sodium nitrite (NaNO_2_).

### Culture of chondrocytes and treatment

Chondrocytes were isolated from pooled femoral and tibial cartilage from individual OA patients by incubating with 1 mg/ml trypsin (Sigma-Aldrich) for 1 h followed by an overnight digestion in 0.5 mg/ml type II collagenase. The following morning, the isolated chondrocytes were washed with complete medium and counted at 1x10^6^ cells/ml.

### Chondrocytes viability

Cells were pipetted (100 μl, 1 × 10^5^ cells/well) into a flat bottom 96-well culture plate and different concentrations of WIN-34B, chlorogenic acid, and mangiferin (10, 40, 100, 200, and 1000 μg/ml) were added in the presence or absence of 10 ng/ml IL-1β. After 48 h incubation at 37°C, 10 μl bromodeoxyuridine (BrdU) was added to each well, and the samples were incubated for 6 h at 37°C. Cells were fixed, anti-BrdU-peroxidase (POD) was added, and then detection was performed using the 3,3^′^,5,5^′^-tetramethylbenzidine (TMB) substrate reaction. The reaction product was quantified using an ELISA reader (480–650 nm).

### Western blot analysis

For Western blot analysis of ADAMTS-4 and MAPK signaling pathways, pieces of cartilage explants culture were immediately frozen in liquid nitrogen and proteins in the resulting powder were extracted with Tris buffer (50 mM Tris, pH 7.0, 100 mM NaCl and 1 μg/ml aprotinin, 0.7 mg/ml pepstatin, 1 mg/ml leupeptin, and 1 mM phenylmethylsulfonyl fluoride) for 12 h. Extract were lyophilized for 2 h to concentrate the proteins, and quantified by the Bio-Rad protein assay. Total protein (20 μg) was separated by electrophoresis by 10% sodium dodecyl sulfate-polyacrylamide gel electrophoresis (SDS-PAGE) and transferred onto a Hybond-C nitrocellulose membrane (Amersham Biosciences, Piscataway, NJ, USA). Blots were blocked in TBS-T containing 5% dry milk for 1 h. Thereafter, blots were probed with a polyclonal antibody against ADAMTS-4 (abcam-ab; Dawinbio, Seoul, Korea; 1:500 dilution), anti-phospho-ERK-1/2, phospho-p38, phospho-JNK, ERK, p38, and JNK (Cell Signaling Technology, Danvers, MA, USA), β-actin or non-immune mouse IgG (Sigma-Aldrich) in blocking buffer at 4°C overnight. Subsequently, each membrane was washed in TBS-T buffer five times for 5 min. Detection was carried out using anti-rabbit hoseradish peroxidase-conjugated IgG (Sigma-Aldrich, 1:5000 dilution) in blocking buffer. Blots were developed by enhanced chemiluminescence (ECL).

For measuring MMP-1, and MMP-13 expression level in IL-1β-stimulated cartilage explants culture, total secreted proteins from 2 ml of conditioned medium were harvested and concentrated by precipitation with trichloroacetic acid (TCA). Proteins (10 μg) were separated by 10% SDS-PAGE. Blots were treated as described above. Membrane were incubated with specific antibodies to MMP-1 and MMP-13 (Calbiochem, San Diego, CA, USA; 1:1000 dilution) in blocking buffer at 4°C overnight, and secondary antibody (Sigma, 1:5000) for 2 h at room temperature. Band intensities were quantified by NIH ImageJ 1.32j (National Institute of Health, USA) software.

### Statistical analyses

Results are expressed as the mean ± SEM. Differences among groups were analyzed by one-way ANOVA followed by Dunnett’s post-hoc test. In the case of two groups, a Student's *t*-test was used. Statistical significance was assessed at *p* < 0.05. Experiments were independently triplicated and results were qualitatively identical. Representative experiments are shown.

## Results

### Effect of WIN-34B on the cytotoxicity of cartilage explants culture and chondrocytes

**Figure 2:**
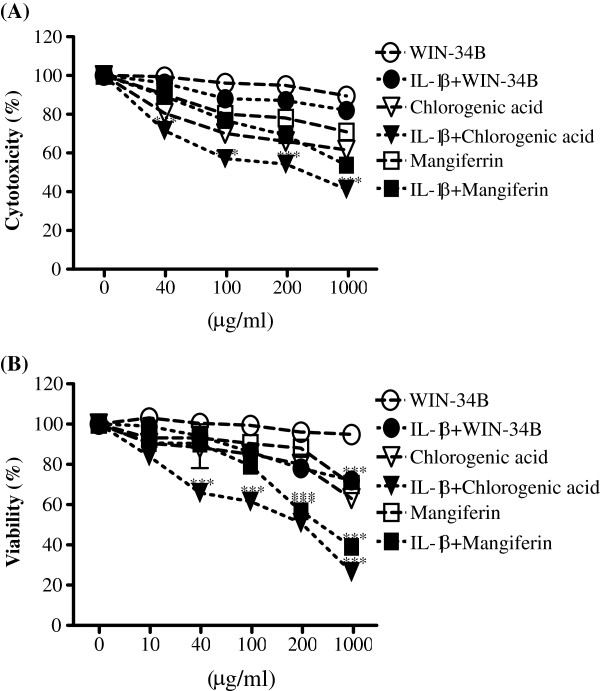
**Effect of WIN**-**34B and its major compounds on the cytotoxicity of IL**-**1β**-**stimulated human cartilage explants culture and chondrocytes.**

WIN-34B was not cytotoxic, as judged by the absence of significant change in LDH activity in the culture medium in the presence or absence of IL-1β. MF did not affect the cytotoxicity of cartilage explants culture in the absence of IL-1β, but a high concentration of MF (1000 μg/ml) was cytotoxic in the presence of IL-1β. CA increased LDH leakage in the culture medium of human OA cartilage explants in the presence or absence of IL-1β (Figure 2A). In chondrocytes, WIN-34B in doses ranging from 0.1–1000 μg/ml did not show the significant effect on the viability of chondrocytes, while viability of IL-1β-stimulated chondrocytes was extent of inhibition (Figure 2B). MF or CA at 1000 μg/ml inhibited the viability by about 30% and 40%, respectively, in the absence of IL-1β, suggesting a possible cytotoxic effect at this concentration (Figure 2B). However, the effect of MF or CA on the viability of chondrocytes did not exceed IC_50_ at concentration up to 200 μg/ml (Figure 2B).

### Effect of WIN-34B on the level of proteoglycan and type II collagen in IL-1β-stimulated cartilage explants culture

**Figure 3:**
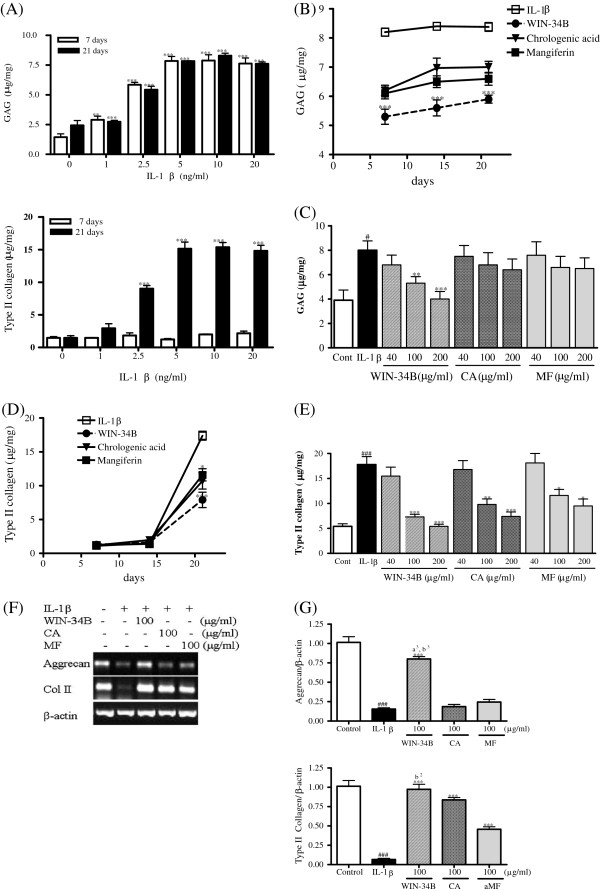
**Effect of WIN**-**34B and its major compounds on cartilage protection of IL**-**1β**-**stimulated human cartilage explants culture.**

**Figure 4:**
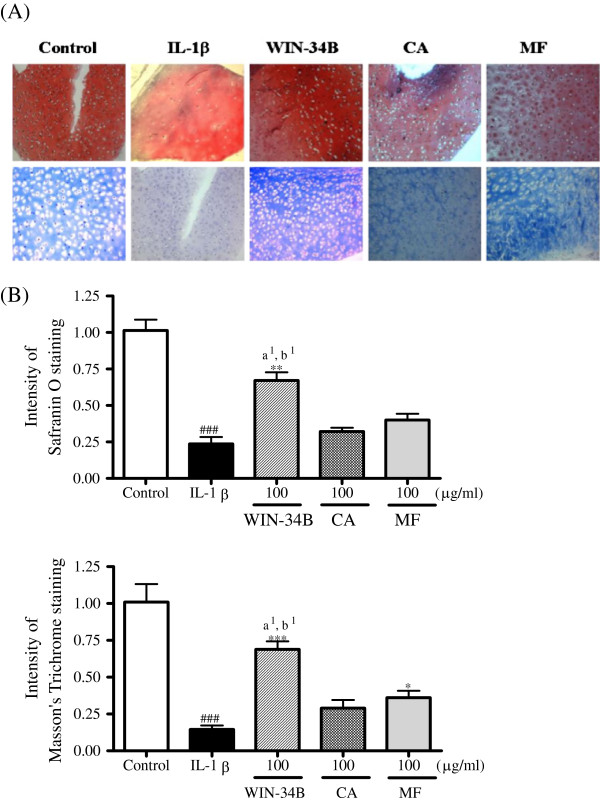
**Histochemical analysis of proteoglycan and collagen in IL**-**1β**-**stimulated human cartilage explants culture.**

In preliminary experiments, to optimize the conditions with which to induce proteoglycan and collagen degradation, articular cartilage was cultured with 1, 2.5, 5, 10, or 20 ng/ml IL-1β for 21 days. These effects were dose-dependent, and 5 ng/ml IL-1β was required to consistently achieve the maximal response. In experimental cultures of cartilage treated with 10 ng/ml IL-1β, more than 7.8 mg/mg of GAG had been released from the tissue after 7 days of culture, and about 7.5 mg/mg after 21 days (Figure 3A), while the release of type II collagen was marginal at any concentration of IL-1β for 7 days, after which there was a marked increase in collagen release to about 75% by 21 days of culture (Figure 3A). WIN-34B, CA, or MF at 100 μg/ml reduced the GAG release until 21 days, but the effect of WIN-34B was superior to CA or MF (Figure 3B). WIN-34B in doses ranging from 40–200 μg/ml significantly inhibited about 28%–49% of the release of GAG at 7 days, while CA and MF displayed no significant differences compared with IL-1β-stimulated cartilage explants culture (Figure 3C). After 21 days, WIN-34B, CA or MF reduced the release of type II collagen in the explants relative to that in IL-1β-treated cultures (Figure 3D), and the degradation of type II collagen was significantly reduced by about 13%–74% by WIN-34B, 11%–62% by CA, and 5%–49% by MF compared with IL-1β treatment in cartilage explants culture (Figure 3E). Also, the mRNA expression of aggrecan and type II collagen was significantly decreased by IL-1β treatment and was almost normalized by WIN-34B at 100 μg/ml IL-1β-stimulated cartilage explants culture (Figures 3F and 3 G). On the contrary, CA and MF were unable to affect the level of aggrecan compared with IL-1β-stimulated cartilage explants culture (Figures 3F and 3 G). The intensity of Safranin O staining was significantly enhanced by about 2.8-fold by WIN-34B at 100 μg/ml compared with IL-1β in cartilage explants culture (Figures 4A and 4B). WIN-34B at 100 μg/ml also significantly enhanced the intensity of Masson’s Trichrome staining by about 5.2-fold compared with IL-1β (Figures 4A and 4C). The difference between WIN-34B and CA or MF was especially pronounced on the contents of proteoglycan and collagen (Figures 4A–4C).

### Effect of WIN-34B on the levels of aggrecanases, MMPs, and TIMPs in IL-1β-stimulated cartilage explants culture

**Figure 5:**
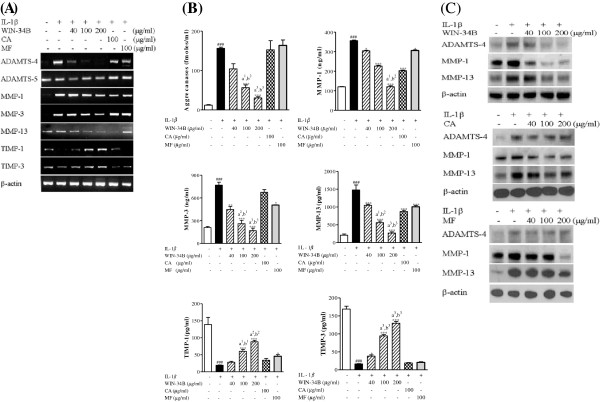
**Effect of WIN**-**34B and its major compounds on the level of matrix proteinases and tissue inhibitor of matrix proteinases in human cartilage explants.**

WIN-34B significantly inhibited the mRNA expression of ADAMTS-4, ADAMTS-5, MMP-1, MMP-3, and MMP-13, and enhanced the mRNA level of TIMP-1 and TIMP-3 in a dose-dependent manner (Figure 5A). WIN-34B in doses from 40–200 μg/ml produced a significant reduction of aggrecanases activity ranging from 32%–80%, but there were no significant changes by CA and MF (Figure 5B). WIN-34B produced a dose-dependent reduction ranging from 15%–66% of MMP-1, 42%–80% of MMP-3, and 32%–86% of MMP-13, while WIN-34B significantly induced production of TIMP-1 (8%–74%) and TIMP-3 (32%–89%) in IL-1β-stimulated cartilage explants culture (Figure 5B). Notably, the reduction of aggrecanases, MMP-1, MMP-3, and MMP-13 by WIN-34B and the enhancement of TIMP-1 and TIMP-3 were superior to the effect seen with CA or MF (Figure 5B). In addition, WIN-34B dose-dependently inhibited the protein expression of ADAMTS-4, MMP-1, and MMP-13 in conditioned medium from IL-1β-stimulated cartilage explants culture. However, CA and MF only slightly inhibited the protein expression of MMP-1 and MMP-13 (Figure 5C).

### Effects of WIN-34B on inflammatory mediators in IL-1β-stimulated cartilage explants culture

**Figure 6:**
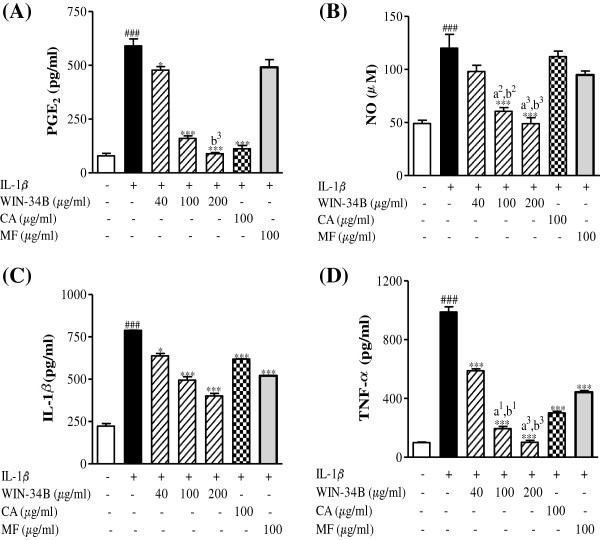
**Effect of WIN**-**34B and its major compounds on the productions of inflammatory mediators in IL**-**1β**-**stimulated cartilage explants culture.**

WIN-34B in doses ranging from 40–200 μg/ml significantly inhibited 16%–83% of PGE_2_ release, 17%–59% of NO production, 21%–51% of IL-1β, and 63%–89% of TNF-α compared with IL-1β − stimulated cartilage explants culture (Figures 6A–6D). CA significantly inhibited the release of PGE_2_ production of IL-1β and TNF-α, while CA was unable to affect the level of NO in IL-1β-stimulated human cartilage explants culture (Figures 6A–6D). MF markedly decreased the production of IL-1β and TNF-α, but did not affect PGE_2_ and NO secretion in IL-1β-stimulated cartilage explants culture (Figures 6A–6D).

### Effect of WIN-34B on phosphorylation of MAPKs in IL-1β-stimulated cartilage explants culture

**Figure 7:**
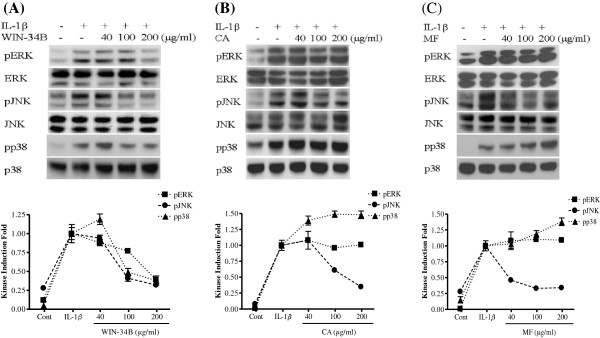
**Effect of WIN**-**34B and its major compounds on pERK**, **pJNK**, **and pp38 MAPK proteins in IL**-**1β**-**stimulated human cartilage explants.**

The phosphorylation of ERK, JNK, and p38 MAPKs was reduced by 25%, 59%, and 63%, respectively, upon treatment with WIN-34B at 100 μg/ml compared with IL-1β − stimulated stimulated cartilage explants culture (Figure 7A). CA and MF dose-dependently inhibited JNK phosphorylation and increased the phosphorylation of p38, while not affecting the phosphorylation of ERK in IL-1β-stimulated cartilage explants culture (Figures 7B and 7C).

## Discussion

A problem in using natural herbal material is the difficulty in standardization of efficacy, which is partially due to factors such as differences in region of origin, harvest period, and cultivation time. Also, for the discovery and development of new treatments, further understanding of the processes leading to disease progression and in particular, the pathways leading to the expression of the MMPs, aggrecanases, and cartilage destruction are of pivotal importance. Therefore, we measured the major components and examined their efficacy to set a standard for use of WIN-34B in practice and in medicine development. In this study, we demonstrated the cartilage-protective effects of WIN-34B compared to standard compounds, CA and MF, in IL-1β-stimulated cartilage explants culture. WIN-34B more effectively improved the cartilage protection without cytotoxicity by modulating MMPs, ADADMTs, TIMPs, and inflammatory mediators, and possibly by inhibiting MAPK pathways.

WIN-34B did not exert cytotoxic effects in the absence of IL-1β in human OA cartilage explants culture and did not affect cell viability in chondrocytes. However, CA was cytotoxic in human cartilage explants culture and chondrocytes.

Previously, we found that even high doses of WIN-34B did not cause toxicity or gastric injury when orally administered to rats [20]. Both single and multiple doses of WIN-34B had no effect on mortality, body weight changes, gross findings, or clinical signs in patients of either sex [19]. This finding was in contrast to those for diclofenac and celecoxib, which cause inflammation and hemorrhage [19,20]. Furthermore, clinical study on patients with OA revealed that WIN-34B not only had a good analgesic efficacy and safety profile, but also showed functional improvements on the time taken to go up and down a standard flight of stairs, duration of morning stiffness, and softening of the affected knee joint. These results support the safety and therapeutic usefulness of WIN-34B for development as an OA treatment.

WIN-34B markedly prevented the release of GAG and type II collagen, which are associated with the down-regulation of matrix proteinases (MMP-1, MMP-3, MMP-13, ADAMTS-4, and ADAMTS-5), inflammatory cytokines (PGE_2_, NO, IL-1β, and TNF-α), and up-regulation of TIMP-1 and TIMP-3 levels in IL-1β-stimulated human OA cartilage culture. However, CA and MF significantly inhibited only collagen release, which is associated with the inhibition of MMP-1, MMP-13, and inflammatory cytokines in IL-1β-stimulated human OA cartilage culture. The inhibition of GAG release and recovery of aggrecan expression by CA and MF was not evident in IL-1β-stimulated human OA cartilage culture. Therefore, we suggest that WIN-34B could be a potential candidate for effective anti-osteoarthritic therapy with cartilage protective properties better than CA or MF.

Protecting ECM components is critical to modifying OA progression and protecting joint functions [21,22]. A number of studies have documented the fact that aggrecan not only resists mechanical loading by enabling the cartilage matrix to attract and imbibe water molecules, but also plays a partial role in preventing collagen degradation in OA pathogenesis [21,23,24]. For this reason, many researchers have investigated the OA-modifying effects of drugs designed to inhibit ADAMTS-4 and ADAMTS-5 [25]. Several studies have reported that glucosamine down-regulates ADAMTS and MMPs including MMP-3, MMP-9, MMP-10, and MMP-12 [25,26]. SKI306X, a commercially-available herbal mixture for OA treatment, inhibits cartilage degradation through the production of MMPs and inflammatory mediators [27]. Inflammatory mediators, such as PGE_2_, NO, IL-1, and TNF-α, play key roles in the progression of cartilage destruction in OA [28,29]. Particularly, IL-1β produces PGE_2_ and NO, and stimulates the expression of other inflammatory cytokines and MMPs [27]. PGE_2_ is a pathologic mediator responsible for the remodeling of cartilage and bone [28]. NO is a pleiotropic mediator involved in the catabolic process of OA, which inhibits the synthesis of proteoglycan and collagen, resulting in the promotion of cartilage destruction [29]. Presently, WIN-34B decreased the level of inflammatory mediators including PGE_2_ and NO, as well as the proinflammatory cytokines, IL-1β, and TNF-α, which are all recognized as inducers of MMPs and aggrecanases. The inhibition of PGE_2_ release, NO production, and TNF-α secretion by WIN-34B was superior to CA or MF. These results suggest that WIN-34B inhibits the pathologic inflammatory molecules in the cartilage destruction of OA.

MAPKs regulate pro-inflammatory cytokine production and downstream signaling cascades leading to catabolic joint destruction [8,30]. Studies in human cells have suggested that MAPK signaling is important for the MMP-derived catabolic response of chondrocytes. Liacini *et al*. showed that TNF-stimulated human OA chondrocytes up-regulated expression of MMP-13, through MAPK 44/42 and Janus-NH_2_-terminal kinase JNK [11]. Similar results in human chondrosarcoma cells supported these findings [31]. Taken together, these findings suggest that MAPK signaling is important for the MMP-derived catabolic response of chondrocytes. Recently reported that intra-articular injections of the p38 MAPK inhibitor SB203580 in the anterior cruciate ligament transection (ACLT) rat model of OA inhibited the expression of MMP-3 and MMP-13 and protected against cartilage damage [32]. Other research showed WIN-34B dose-dependently diminished phosphorylation of ERK, JNK, and p38 MAPK, as well as MMPs and aggrecanases in IL-1β-stimulated cartilage explants culture. However, CA and MF increased phosphorylation of p38 and suppressed phosphorylation of JNK, but did not affect the phosphorylation of ERK. Inhibition of the MAPK P44/42 pathway by either U0126 or PD98059 leads to abrogation of the expression and activity of MMPs and aggrecanases and ADAMTS. Inhibitors of the p38 MAPK and JNK pathways were also investigated by SB203580 or SB202190 and PP1, respectively. However, inhibition of these pathways resulted in inhibition of MMP expression and activity, but did not influence aggrecanases activity [33]. The current line of investigations suggests that p38 MAPK and JNK activity could be associated with MMP-mediated irreversible cartilage damage, whereas the processes needed for normal repair mechanism and aggrecanase activity may in part be controlled by MAPK p44/42-activities [33,34]. From these results we can suggest that WIN-34B may be critical role on cartilage protection and anti-inflammatory effect by the downregulation of pERK, p38 MAPK and pJNK signaling pathways.

## Conclusions

WIN-34B has cartilage protective effects similar to or better than its standard compound CA or MF through the regulation of matrix proteinases (aggrecanases and MMPs/TIMPs), inflammatory mediators (PGE_2_, NO, IL-1β, and TNF-α) and the MAPK pathways. These results suggest that WIN-34B could be a potential candidate for effective anti-osteoarthritic therapy with cartilage protective properties and without toxicity instead of existing OA treatment.

## Competing interests

The authors declare that they have no competing interests.

## Authors’ contributions

DSP was project supervisor and proofread the manuscript. JEH conducted the cartilage explants culture, participated in designing the experimental details, wrote the manuscript, and critically revising the paper. BKS carried out the histological experiments, analyzed the data, and wrote the manuscript. YHB, SL, JDL, DYC participated in the study design, data interpretation, and corrected the manuscript for publication. All authors read and approved the final manuscript.

## Pre-publication history

The pre-publication history for this paper can be accessed here:

http://www.biomedcentral.com/1472-6882/12/256/prepub
